# Supplementary material for: Modelling Skylarks (Alauda arvensis) to Predict Impacts of Changes in Land Management and Policy: Development and Testing of an Agent-Based Model
Source: PLoS One. 2013 Jun 6;8(6):e65803. doi: 10.1371/journal.pone.0065803 (PMC3675089; doi:10.1371/journal.pone.0065803)
Supplement: Supporting Information S4 — The skylark ODdox as a zipped archive. (ZIP) [file pone.0065803.s004.zip › Skylark_ODdox/globals_0x65.html]

ALMaSS Skylark ODdox: File Members


ALMaSS Skylark ODdox
 2.0


- Main Page
- Related Pages
- Classes
- Files

- File List
- File Members

- All
- Functions
- Variables
- Typedefs
- Enumerations
- Enumerator
- Macros

- \_
- a
- b
- c
- d
- e
- f
- g
- h
- i
- j
- k
- l
- m
- n
- o
- p
- r
- s
- t
- u
- v
- w
- x

Here is a list of all file members with links to the files they belong to:

### - e -

- EggTemp
  : skylarks\_all.cpp
- EL\_BUG\_GLUE
  : elements.cpp
- EL\_BUG\_PERCENT\_A
  : elements.cpp
- EL\_BUG\_PERCENT\_B
  : elements.cpp
- EL\_BUG\_PERCENT\_C
  : elements.cpp
- EL\_BUG\_PERCENT\_D
  : elements.cpp
- EL\_BUG\_PERCENT\_Edges\_A
  : elements.cpp
- EL\_BUG\_PERCENT\_Edges\_B
  : elements.cpp
- EL\_BUG\_PERCENT\_Edges\_C
  : elements.cpp
- EL\_BUG\_PERCENT\_G\_A
  : elements.cpp
- EL\_BUG\_PERCENT\_G\_B
  : elements.cpp
- EL\_BUG\_PERCENT\_G\_C
  : elements.cpp
- EL\_BUG\_PERCENT\_SB\_A
  : elements.cpp
- EL\_BUG\_PERCENT\_SB\_B
  : elements.cpp
- EL\_BUG\_PERCENT\_SB\_C
  : elements.cpp
- EL\_BUG\_PERCENT\_WR\_A
  : elements.cpp
- EL\_BUG\_PERCENT\_WR\_B
  : elements.cpp
- EL\_BUG\_PERCENT\_WR\_C
  : elements.cpp
- EL\_BUG\_PERCENT\_WRy\_A
  : elements.cpp
- EL\_BUG\_PERCENT\_WRy\_B
  : elements.cpp
- EL\_BUG\_PERCENT\_WRy\_C
  : elements.cpp
- EL\_BUG\_PERCENT\_WW\_A
  : elements.cpp
- EL\_BUG\_PERCENT\_WW\_B
  : elements.cpp
- EL\_BUG\_PERCENT\_WW\_C
  : elements.cpp
- EL\_BUG\_SLOPE
  : elements.cpp
- EL\_GROWTH\_DATE\_MAGIC
  : elements.cpp
- EL\_GROWTH\_DAYDEG\_MAGIC
  : elements.cpp
- EL\_GROWTH\_PHASE\_SHIFT\_LEVEL
  : elements.cpp
- EL\_HERBICIDE\_DELAYTIME
  : elements.h
- EL\_MAX\_USERSPACE
  : elements.h
- EL\_PLANT\_BIOMASS
  : elements.cpp
- EL\_STRIGLING\_DELAYTIME
  : elements.h
- EL\_TRAMLINE\_DECAYTIME
  : elements.h
- EL\_VEG\_HEIGHTSCALE
  : elements.cpp
- EL\_VEG\_START\_LAIT
  : elements.cpp
- EL\_WEED\_GLUE
  : elements.cpp
- EL\_WEED\_PERCENT
  : elements.cpp
- EL\_WEED\_SLOPE
  : elements.cpp
- EM\_coldblood1
  : skylarks\_all.cpp
- EM\_coldblood2
  : skylarks\_all.cpp
- EM\_nest\_T
  : skylarks\_all.cpp
- EM\_warmblood1
  : skylarks\_all.cpp
- EM\_warmblood2
  : skylarks\_all.cpp
- ExtraBroodHeat
  : skylarks\_all.cpp


- Generated on Thu Jan 10 2013 13:15:39 for ALMaSS Skylark ODdox by
   1.8.1.1
